# MiR-106a directly targets LIMK1 to inhibit proliferation and EMT of oral carcinoma cells

**DOI:** 10.1186/s11658-018-0127-8

**Published:** 2019-03-06

**Authors:** Bingxia Shi, Chao Ma, Guolin Liu, Yanjun Guo

**Affiliations:** 1 Oral and Maxillofacial Surgery, Cangzhou Central Hospital, No. 16 Xinhua West Road, Hebei, 061000 People’s Republic of China; 2 Department of Medical Plastic Surgery, Cangzhou Central Hospital, Hebei, 061000 People’s Republic of China

**Keywords:** Oral squamous carcinoma, MicroRNA-106a, LIM kinase 1, Proliferation, Epithelial–mesenchymal transition

## Abstract

**Background:**

LIM kinase 1 (LIMK1) expression levels are closely associated with microRNA (miRNA) processing. Higher levels of LIMK1 are reported during the progression of many cancers. Our study explored the interaction between LIMK1 and miR-106a in oral squamous cell carcinoma (OSCC).

**Methods:**

Quantitative RT-PCR was performed to detect the levels of LIMK1 and miR-106a in OSCC tissues and cell lines. The rates of cell proliferation and epithelial–mesenchymal transition (EMT) were assessed to determine the biological functions of miR-106a and LIMK1 in OSCC cells. The mRNA and protein levels of LIMK1 were measured using quantitative RT-PCR and western blotting. Luciferase assays were performed to validate LIMK1 as an miR-106a target in OSCC cells.

**Results:**

We found that the level of miR-106a significantly decreased and the expression of LIMK1 significantly increased in OSCC tissues and cell lines. There was a close association between these changes. Knockdown of LIMK1 significantly inhibited the proliferation and EMT of OSCC cells. The bioinformatics analysis predicted that LIMK1 is a potential target gene of miR-106a and the luciferase reporter assay confirmed that miR-106a could directly target LIMK1. Introduction of miR-106a to OSCC cells had similar effects to LIMK1 silencing. Overexpression of LIMK1 in OSCC cells partially reversed the inhibitory effects of the miR-106a mimic.

**Conclusion:**

MiR-106a inhibited the cell proliferation and EMT of OSCC cells by directly decreasing LIMK1 expression.

## Background

Oral squamous cell carcinoma (OSCC) is a malignant tumor of the oral maxillofacial region [1, 2]. It has a high incidence rate. Despite recent advances in both clinical and experimental fields, the prognosis is still unfavorable due to its invasive characteristics and highly malignancy. The 5-year survival rates remain at less than 50% and have not been improved in the last 3 decades [3–5]. Traditional treatment methods have been unable to meet patient needs, so new therapeutic strategies must be evaluated. Increasingly, research is focusing on the pathogenesis of tumor-targeted therapy and gene research: the role of genes involved in tumorigenesis and metastasis; the molecular mechanisms of those processes; and the targeting of specific genes. It is vital to uncover the biological mechanisms of cancers to ensure the correct identification of useful biomarkers and novel therapeutic targets.

LIM kinase-1 (LIMK1) and LIM kinase-2 (LIMK2) belong to a small subfamily with a unique combination of 2 N-terminal LIM motifs and a C-terminal protein kinase domain. LIMK1, a serine/threonine kinase, regulates actin polymerization via phosphorylation and inactivation of the actin-binding factor cofilin (CFL1) [6], which is a critical regulator in processes including cell movement and the cell cycle [7, 8]. Cancer tumorigenesis and metastasis are affected when activated LIMK1 phosphorylates CFL1 [9]. The role of LIMK1 in OSCC is still unknown.

MicroRNAs (miRNAs) are a new class of endogenous, short, small, single-stranded, conserved RNAs that regulate gene expression by binding to the 3′-untranslated region (3’-UTR) of their target messenger RNAs (mRNAs) [10–12]. A growing body of research has showed that miRNAs play an important role in many biological processes such as cell development, invasion, proliferation, differentiation, metabolism, apoptosis and migration [13–16]. There is also increasing evidence that dysregulated expression of miRNA is related to tumor initiation, development and cancer death through regulating tumor inhibitor gene or oncogene [16–18]. However, the effects of miR-106a in OSCC remain unclear.

In this study, to explore the role of miR-106a in OSCC, we determined the expression of LIMK1 in OSCC tissues and cell lines. Using the online database TargetScan 7.2, we predicted that miR-106a might directly target LIMK1. We also investigated the relationship between LIMK1 and miR-106a in OSCC tissues. Finally, we studied the effects of LIMK1 silencing or miR-106a overexpression on OSCC cell invasion and epithelial–mesenchymal transition (EMT).

## Materials and methods

### Human tissue samples

Human OSCC tissues (*n* = 20) and their adjacent non-cancerous tissues (*n* = 10) were collected from patients at the Cangzhou Central Hospital between May 2015 and May 2017. All samples were immediately frozen in liquid nitrogen for subsequent quantitative RT-PCR analysis. This study was approved by the Ethical Committee of Cangzhou Central Hospital (CZCH2015052609) and complied with the guidelines and principles of the Declaration of Helsinki. All participants signed written informed consent.

### Cell culture

The OSCC cell lines SCC1, Cal-27 and SCC4 and a normal oral keratinocyte cell line (NHOK) were purchased from the American Type Culture Collection (ATCC). All the cells were cultivated in DMEM/F12 medium supplemented with heat-inactivated 10% FBS (GIBCO) and penicillin/streptomycin (100 U/ml and 100 mg/ml, respectively) at 37 °C in a humidified atmosphere of 5% CO_2_.

### Transient transfection

The miR-106a mimics, miR-106a inhibitors, negative control (NC), siRNA for LIMK1 (si-LIMK1) and siRNA-negative control (si-NC) were synthesized and purified by Gene-Pharma. The LIMK1-overexpression plasmid was generated by inserting LIMK1 cDNA into a pcDNA3.1 vector. This plasmid was sequenced and confirmed by Gene-Pharma. The miR-106a mimics, miR-106a inhibitors, si-LIMK1 and LIMK1-overexpression plasmid were transfected using Lipofectamine 3000 reagent (Invitrogen) per the manufacturer’s protocols. Cells (10^7^/well) were transfected for 48 h in a 6 well-plate, and total RNA and protein were collected.

### RNA extraction and quantitative real-time PCR

Total RNA was extracted from tissues and cells using Trizol reagent (Invitrogen) per the manufacturer’s protocol. Reverse transcription was performed using the miScript Reverse Transcription Kit (QIAGEN). The QuantiTect SYBR Green RT-PCR Kit (QIAGEN) was used with the ABI 7500 Fast Real-Time PCR System (Applied Biosystems) for quantitative real-time PCR analysis following the manufacturer’s instructions. Denaturation was performed at 94 °C for 1 min, annealing at 59 °C for 1 min, and elongation at 72 °C for 1 min for 32 cycles, followed by 72 °C for 10 min. The relative expression levels of miR-106a, LIMK1, N-cadherin, E-cadherin and vimentin were normalized to those of the internal control U6 or GAPDH using the comparative delta C_T_ (2^-ΔΔCT^) method. Each sample was analyzed in triplicate. Prime sequences are shown in Table 1.

**Table 1:** Sequence of primers for qRT-PCR

Gene	Primer Sequence
LIMK1	F: 5’-CAAGGGACTGGTTATGGTGGC-3′
	R: 5’-CCCCGTCACCGATAAAGGTC-3’
LIMK2	F: 5’-GGATTCCCTCACCAACTGGTA-3’
	R: 5’-AGCCACCATAAAAGGCCCTG-3’
E-cadherin	F: 5’-TACACTGCCCAGGAGCCAGA-3’
	R: 5’-TGGCACCAGTGTCCGGATTA-3’
N-cadherin	F: 5′- TCAGGCGTCTGTAGAGGCTT-3’
	R: 5′- ATGCACATCCTTCGATAAGACTG-3’
Vimentin	F: 5’-GACGCCATCAACACCGAGTT-3’
	R: 5’-CTTTGTCGTTGGTTAGCTGGT-3’
Snail	F: 5’-TCGGAAGCCTAACTACAGCGA-3’
	R: 5’-AGATGAGCATTGGCAGCGAG-3’
Slug	F: 5’-CGAACTGGACACACATACAGTG-3’
	R: 5’-CTGAGGATCTCTGGTTGTGGT-3’
ZEB1	F: 5’-GATGATGAATGCGAGTCAGATGC-3’
	R: 5’-ACAGCAGTGTCTTGTTGTTGT-3’
PCNA	F: 5’-CCTGCTGGGATATTAGCTCCA-3’
	R: 5’-CAGCGGTAGGTGTCGAAGC-3’
CDK2	F: 5’-TGTTTAACGACTTTGGACCGC-3’
	R: 5’-CCATCTCCTCTATGACTGACAGC-3’
CDK4	F: 5’-GGGGACCTAGAGCAACTTACT-3’
	R: 5’-CAGCGCAGTCCTTCCAAAT-3’
cyclin D1	F: 5’-GCTGCGAAGTGGAAACCATC-3’
	R: 5’-CCTCCTTCTGCACACATTTGAA-3’
cyclin E1	F: 5’-AAGGAGCGGGACACCATGA-3’
	R: 5’-ACGGTCACGTTTGCCTTCC-3’
p21	F: 5’-TGTCCGTCAGAACCCATGC-3’
	R: 5’-AAAGTCGAAGTTCCATCGCTC-3’
p27	F: 5’-AACGTGCGAGTGTCTAACGG-3’
	R: 5’-CCCTCTAGGGGTTTGTGATTCT-3’
ZEB2	F: 5’-CAAGAGGCGCAAACAAGCC-3’
	R: 5’-GGTTGGCAATACCGTCATCC-3’
U6	F: 5’-CTCGCTTCGGCAGCACA-3’
	R: 5’-AACGCTTCACGAATTTGCGT-3’
GAPDH	F: 5’-GAGTCAACGGATTTGGTCGTATTG-3’
	R: 5’-CCTGGAAGATGGTGATGGGATT-3’

### Protein extraction and western blot analysis

Transfected cells were solubilized with RIPA lysis buffer (Beyotime Biotechnology) containing protease inhibitors (Millipore). The protein concentration was measured using a BCA protein assay kit (Beyotime Biotechnology). Equal amounts of protein were separated with 12% SDS-PAGE and transferred to polyvinylidene difluoride (PVDF) membranes (Millipore). The membranes were then blocked with 5% non-fat milk in TBST for 1 h at room temperature, followed by incubation with Abcam primary antibodies for LIMK1 (ab81046), E-cadherin (ab76055), N-cadherin (ab18203), vimentin (ab92547), SNAIL (ab53519), SLUG (ab51772) and ZEB1 (ab203829) overnight at 4 °C. Subsequently, the membranes were washed three times with TBST and probed with the corresponding horseradish peroxidase-conjugated secondary antibodies (Cell Signaling Technology Inc.) for 2 h at room temperature. ECL reagent (Pierce) was used to detect the signals on the membranes.

### Luciferase reporter assay

The luciferase reporter vectors (pGL3-LIMK1–3’UTR WT and pGL3-LIMK1–3’UTR MUT) were synthesized by GenePharma. SCC4 cells were seeded into 24-well plates and transfected with pGL3-LIMK1–3’UTR WT or pGL3-LIMK1–3’UTR MUT, along with miR-106a mimics or miR-NC using Lipofectamine 2000 per the manufacturer’s instructions. After transfection for 48 h, luciferase reporter assays were performed with the Promega Dual-Luciferase Reporter Assay System. The relative firefly luciferase activities were measured via normalization to renilla luciferase activities.

### Statistical analysis

The data are expressed as the means ± standard error of the mean (SEM). The number of independent experiments is represented by “n”. The relationship between miR-106a and the clinicopathological characteristics was tested using the chi-square test. Correlations between miR-106a and LIMK1 mRNA levels were analyzed using Pearson’s correlation coefficient. Multiple comparisons were performed using one-way ANOVA followed by Tukey’s multiple-comparison test. Two-tailed Student’s *t*-test was used for other comparisons. *p* < 0.05 was considered statistically significant.

## Results

### High expression of LIMK1 correlates with low levels of miR-106a in OSCC tissues and cells

**Fig. 1:**
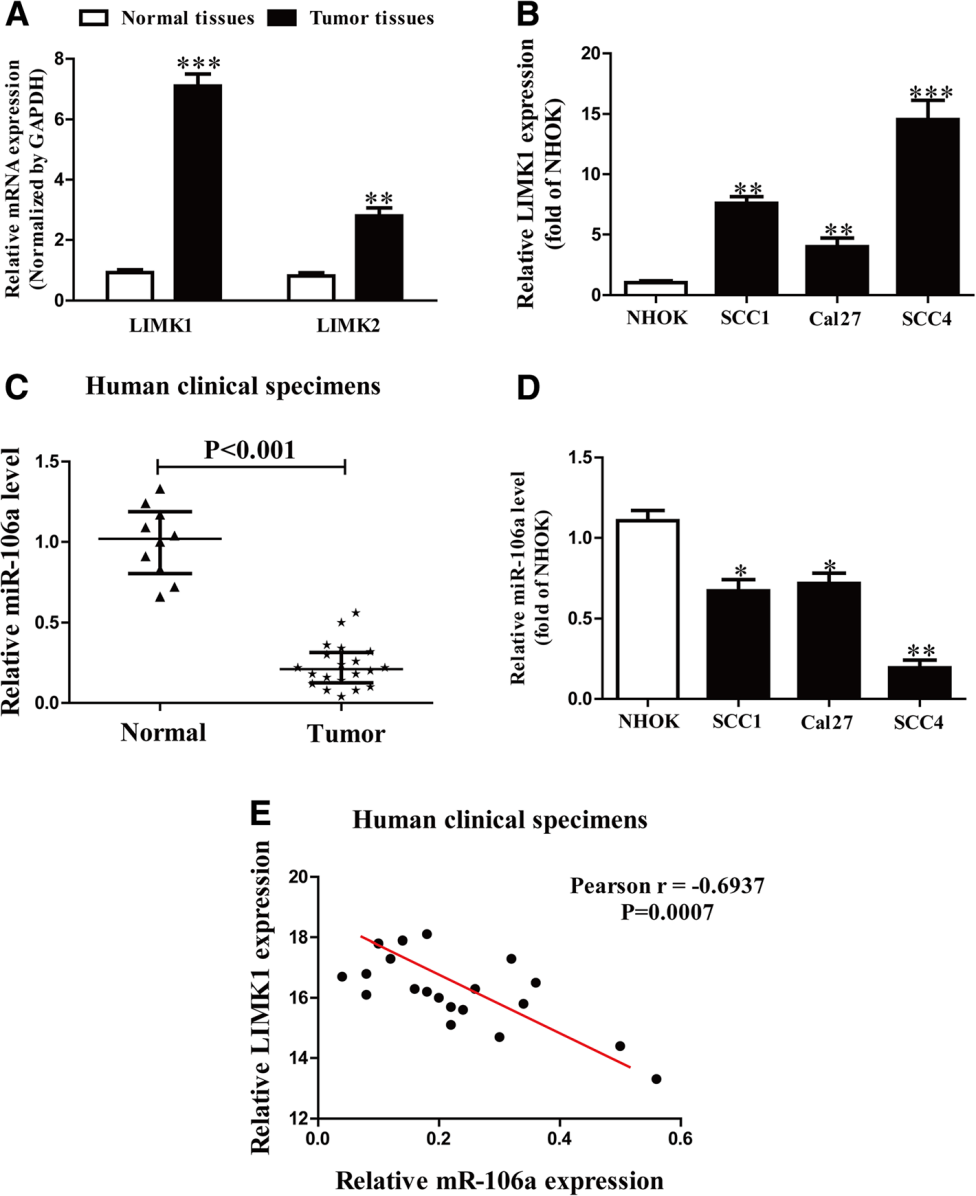
The expressions of LIMK1 and miR-106a in OSCC tissues and cell lines. **a** Quantitative RT-PCR analysis of LIMK1 and LIMK2 expressions in OSCC tissues (*n* = 20) and adjacent normal tissues (*n* = 10). Transcript levels were normalized to GAPDH expression. **b** Relative LIMK1 expression analyzed via quantitative RT-PCR in three OSCC cell lines normalized to GAPDH (*n* = 6). **c** Quantitative RT-PCR analysis of miR-106a level in OSCC tissues and adjacent normal tissues. Transcript levels were normalized to U6. **d** Relative miR-106a level analyzed via quantitative RT-PCR in three OSCC cell lines normalized to U6 (*n* = 6). **e** Pearson’s correlation analysis of the relative expression levels of miR-106a and the relative LIMK1 mRNA levels in OSCC tissues. All data are presented as means ± SEM. *p < 0.05, **p < 0.01, ***p < 0.001 vs. normal tissues or NHOK

The levels of LIMK1 and LIMK2 were determined in OSCC tissues using quantitative RT-PCR. The results showed that the mRNA level of LIMK1 was higher than that of LIMK2, and higher in OSCC tissues than in adjacent tissues (Fig. 1a).

We also determined the mRNA level of LIMK1 in three OSCC cell lines (SCC1, Cal27 and SCC4) and a human normal oral keratnocyte cell culture (NHOK). The level of LIMK1 in SCC4 cells was higher than that in the other two OSCC cell lines or in NHOK (Fig. 1b).

Using the online database microRNA.org, we found that miR-106a may directly target LIMK1. Our findings demonstrated that the level of miR-106a in the OSCC tissues was significantly lower than in the adjacent tissues (Fig. 1c). We also confirmed that miR-106a expression was lower in SCC4 cells than that in the other four OSCC cell lines (Fig. 1d). SCC4 cells were used in the following experiments.

Pearson’s correlation analysis was performed to determine whether the expression of LIMK1 was associated with miR-106a in OSCC. It revealed a significant inverse correlation between LIMK1 and miR-106a in OSCC tissues (Fig. 1e). Based on these data, we predicted that LIMK1 might be negatively regulated by miR-106a.

### Knockdown of LIMK1 inhibited cell proliferation and EMT of OSCC cells

**Fig. 2:**
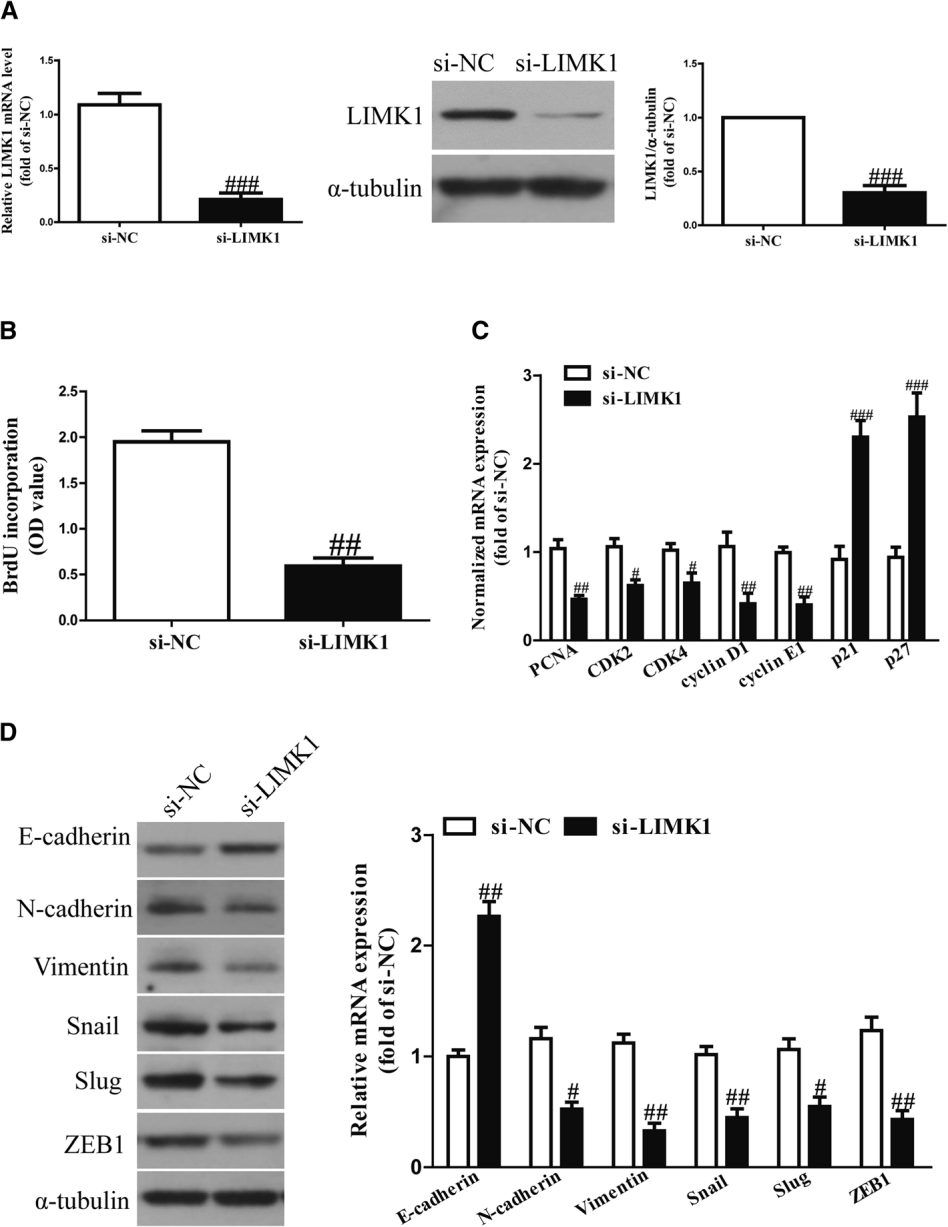
The effects of LIMK1 silencing on the proliferation and EMT in OSCC cells. SCC4 cells were transfected with si-LIMK1 or si-NC for 48 h. **a** The mRNA and protein expressions of LIMK1 were determined via quantitative RT-PCR and western blot, respectively. **b** Cell proliferation was assessed with a BrdU-ELISA assay. **c** The mRNA expressions of PCNA, CDK2, CDK4, cyclin D1, cyclin E1, p21 and p27 were determined via quantitative RT-PCR. **d** The expressions of E-cadherin, N-cadherin, vimentin, SNAIL, SLUG and ZEB1 were determined via quantitative RT-PCR and western blot assays, respectively. All data are presented as means ± SEM, *n* = 6. ^#^*p* < 0.05, ^##^*p* < 0.01 vs. si-NC

To explore the functional roles of LIMK1 in OSCC cells, SCC4 cells were transfected with siRNA-NC or siRNA-LIMK1 for 48 h. After transfection, the proliferation and EMT of OSCC cells were assessed. Western blot analysis showed that the LIMK1 expression had significantly decreased in SCC4 cells transfected with siRNA-LIMK1 (si-LIMK1) for 48 h compared to the siRNA-NC (si-NC) group (Fig. 2a). The Brdu-ELISA assay indicated that knockdown of LIMK1 could significantly inhibit the proliferation of SCC4 cells (Fig. 2b), and the qRT-PCR assay showed that downregulation of LIMK1 decreased the mRNA levels of PCNA, CDK2, CDK4, cyclin D1 and cyclin E1 and increased the mRNA levels of p21 and p27 (Fig. 2c). Furthermore, knockdown of LIMK1 dramatically enhanced the expression of the epithelial marker E-cadherin and reduced the expressions of the mesenchymal markers N-cadherin and vimentin in SCC4 cells (Fig. 2d).

We also evaluated the expressions of EMT-related transcription factors in SCC4 cells. Silencing LIMK1 expression significantly decreased the mRNA and protein expressions of SNAIL, SLUG and ZEB1 in SCC4 cells (Fig. 2d). These results show that LIMK1 silencing significantly inhibited the proliferation and EMT of OSCC cells.

### MiR-106a directly targeted LIMK1 3’UTR

**Fig. 3:**
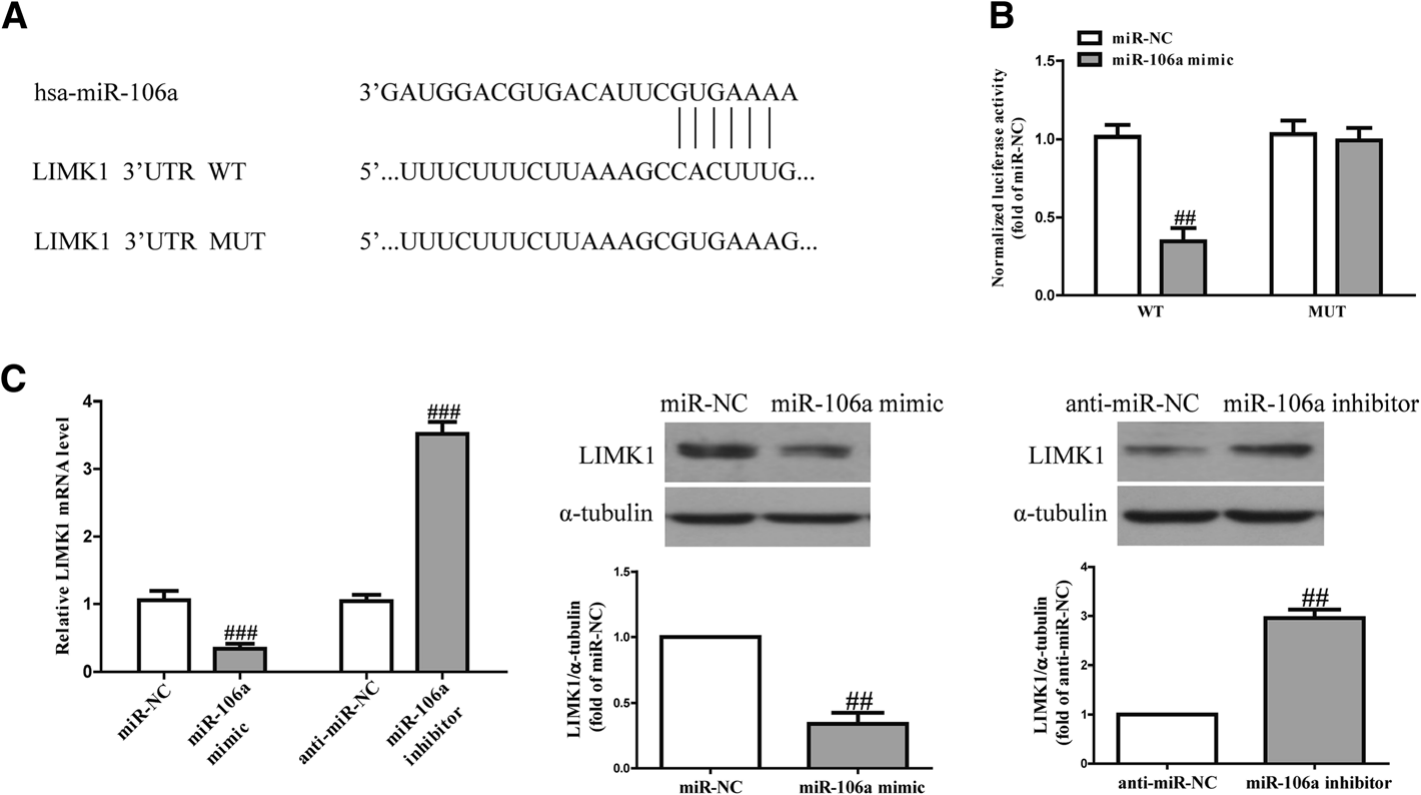
LIMK1 is a direct target of miR-106a. SCC4 cells were transfected with an miR-106a mimic or inhibitor for 48 h. **a** Schematic representation of LIMK1 3’UTRs showing the putative miRNA target site. **b** The analysis of the relative luciferase activities of LIMK1-WT and LIMK1-MUT. **c** The mRNA and protein expressions of LIMK1 were determined via quantitative RT-PCR and western blot, respectively. All data are presented as means ± SEM, n = 6. ^##^p < 0.01, ^###^p < 0.001 vs. miR-NC or anti-miR-NC

Using the TargetScan 7.2 online database, we identified a miR-106a-binding site in the 3’UTR of LIMK1 (Fig. 3a). To validate whether LIMK1 is a direct target of miR-106a, luciferase plasmids containing the potential LIMK1 miR-106a-binding sites (WT) or a mutated LIMK1 3’UTR were constructed (Fig. 3a). Overexpression of miR-106a inhibited WT LIMK1 reporter activity but not the activity of the mutated reporter construct in SCC4 cells, demonstrating that miR-106a could specifically target the LIMK1 3’UTR by binding to the seed sequence (Fig. 3b). Next, we confirmed the results at the mRNA and protein levels. Introduction of miR-106a significantly decreased the expression of LIMK1, whereas knockdown of miR-106a increased the LIMK1 expression in SCC4 cells (Fig. 3c). These data indicate that miR-106a directly regulated LIMK1 expression in OSCC cells through 3’UTR sequence binding.

### The effect of miR-106a on the proliferation of OSCC cells

**Fig. 4:**
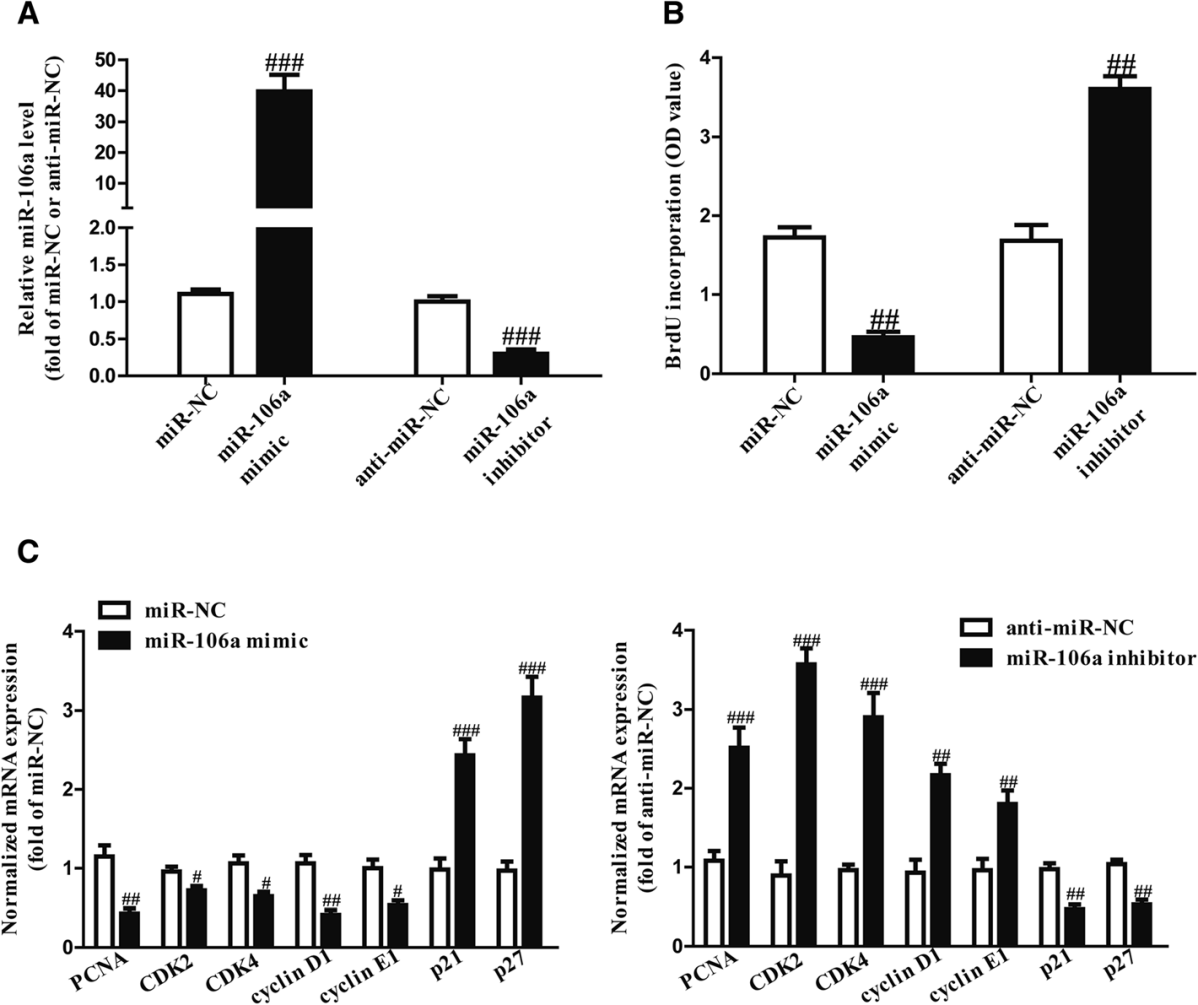
The effects of miR-106a on proliferation and related molecules in OSCC cells. SCC4 cells were transfected with the miR-106a mimic or inhibitor for 48 h. **a** The level of miR-106a was detected via quantitative RT-PCR. **b** Cell proliferation was assessed with a BrdU-ELISA assay. **c** The mRNA expressions of PCNA, CDK2, CDK4, cyclin D1, cyclin E1, p21 and p27 were determined via quantitative RT-PCR. All data are presented as means ± SEM, n = 6. ^#^p < 0.05, ^##^p < 0.01, ^###^p < 0.001 vs. miR-NC or anti-miR-NC

After transfection with a miR-106a mimic or inhibitor, the qRT-PCR analysis showed that the level of miR-106a was respectively significantly upregulated or downregulated compared to the miR-NC group (Fig. 4a), respectively. The results from Brdu-ELISA assay indicated that introduction of miR-106a markedly inhibited the proliferation of SCC4 cells (Fig. 4b). However, cell proliferation was promoted compared with the miR-NC group in SCC4 cells transfected with a miR-106a inhibitor (Fig. 4b).

To further confirm these results, we tested the effects of miR-106a on several proliferation- and cell cycle-related genes. As shown in Fig. 4c, the overexpression of miR-106a decreased the mRNA levels of PCNA, CDK2, CDK4, cyclin D1 and cyclin E1 and increased the mRNA levels of p21 and p27 in SCC4 cells. The knockdown of miR-106a had opposite effects to those of the miR-106a mimic (Fig. 4c).

### The effects of miR-106a on EMT in OSCC cells

**Fig. 5:**
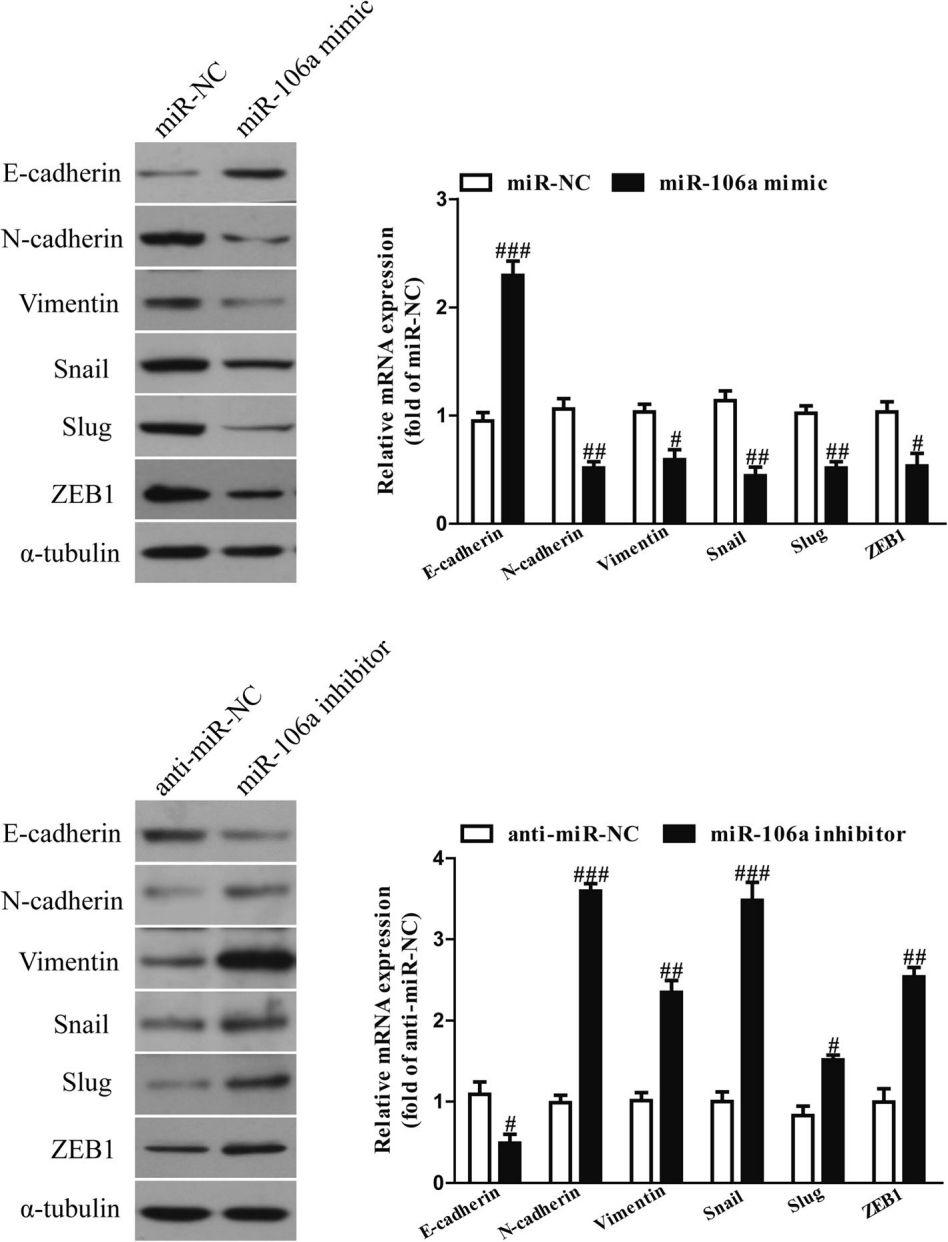
The effects of miR-106a on the expressions of EMT-related molecules in OSCC cells. SCC4 cells were transfected with an miR-106a mimic or inhibitor for 48 h. The mRNA and protein expressions of E-cadherin, N-cadherin, vimentin, SNAIL, SLUG and ZEB1 were determined via quantitative RT-PCR and western blot, respectively. All data are presented as means ± SEM, n = 6. ^#^p < 0.05, ^##^p < 0.01, ^###^p < 0.001 vs. miR-NC or anti-miR-NC

For further study, we examined the effects of miR-106a on the expressions of EMT markers at the mRNA and protein levels in OSCC cells. Overexpression of miR-106a dramatically enhanced the expression of E-cadherin and reduced the expressions of N-cadherin and vimentin in SCC4 cells (Fig. 5). However, the miR-106a inhibitor had the opposite effects on the expressions of these EMT markers (Fig. 5). Moreover, we also determined the expressions of EMT-related transcription factors in SCC4 cells after transfection with a miR-106a mimic or inhibitor. Increasing the miR-106a level significantly decreased the mRNA and protein expressions of SNAIL, SLUG and ZEB1 in SCC4 cells (Fig. 5). However, the knockdown of miR-106a significantly increased the mRNA and protein expressions of SNAIL, SLUG and ZEB1 in SCC4 cells (Fig. 5). Our data suggest that miR-106a upregulation significantly inhibited the EMT of OSCC cells. Consequently, miR-106a overexpression had similar effects to LIMK1 silencing on OSCC cells.

### Overexpression of LIMK1 markedly reversed the effects of miR-106a upregulation on the proliferation and EMT of OSCC cells

**Fig. 6:**
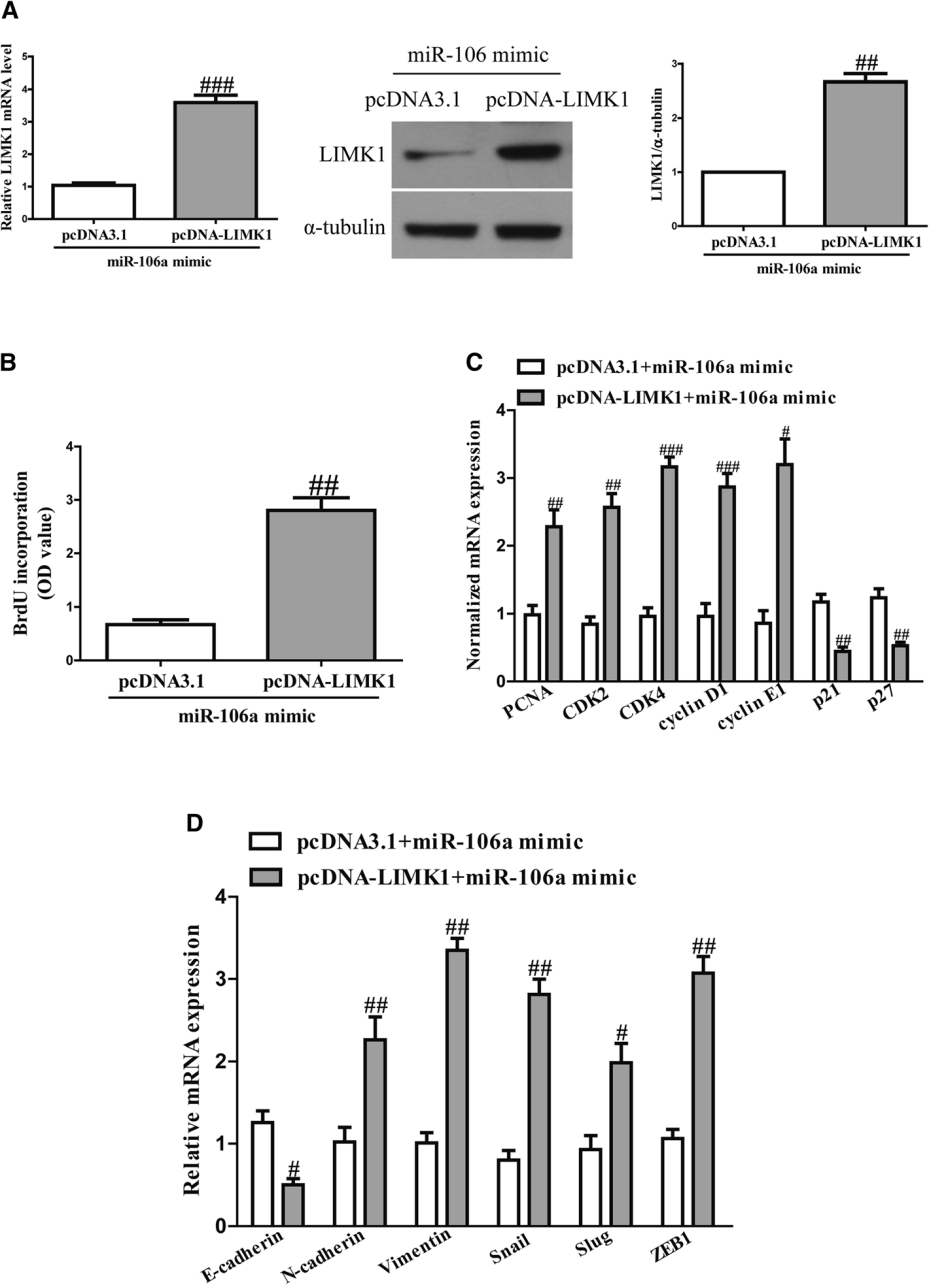
Introduction of LIMK1 partially promoted cell proliferation and EMT in miR-106a-overexpressing OSCC cells. SCC4 cells were transfected with an miR-106a mimic with or without pcDNA-LIMK1 vector. **a** The mRNA and protein expressions of LIMK1 were determined via quantitative RT-PCR and western blot assays, respectively. **b** Cell proliferation was assessed using a BrdU-ELISA assay. **c** The mRNA expressions of PCNA, CDK2, CDK4, cyclin D1, cyclin E1, p21 and p27 were determined using quantitative RT-PCR. **d** The expressions of E-cadherin, N-cadherin, vimentin, SNAIL, SLUG and ZEB1 were determined via quantitative RT-PCR. All data are presented as means ± SEM, n = 6. ^#^*p* < 0.05, ^##^*p* < 0.01, ^###^*p* < 0.001 vs. pcDNA3.1 + miR-106amimic

To determine whether miR-106a targeting LIMK1 was responsible for inhibition of the proliferation and EMT of OSCC cells, we constructed an expression vector that encoded the entire LIMK1 coding sequence but lacked the 3’-UTR. Then, we co-transfected this vector (pcDNA-LIMK1) or its negative control (pcDNA3.1) with the miR-106a mimic or miR-NC into SCC4 cells (Fig. 6a). Cell proliferation assay data showed that concomitant overexpression of miR-106a and LIMK1 abrogated the inhibitory effect of the miR-106a mimic (Fig. 6b). Meanwhile, the mRNA levels of PCNA, CDK2, CDK4, cyclin D1 and cyclin E1 increased and the mRNA levels of p21 and p27 decreased in miR-106a-overexpressing SCC4 cells after exogenous introduction of LIMK1 (Fig. 6c).

Increased LIMK1 expression promoted the EMT of SCC4 cells transfected with the miR-106a mimic (Fig. 6d). Therefore, the inhibitory effects of miR-106a were reversed by LIMK1 overexpression. These results clearly confirm that miR-106a inhibited the proliferation and EMT of OSCC cells by directly downregulating LIMK1 expression, and miR-106a targeting LIMK1 was responsible for inhibiting the proliferation and EMT of OSCC cells.

## Discussion

Many studies support an oncogenic role for LIMK1 in colorectal cancer, lung cancer, prostate cancer and osteosarcoma [19–22]. Liao et al. reported that LIMK1 played an important role in promoting colorectal cancer progression [19]. Knockdown of LIMK1 inhibits migration of lung cancer cells and enhances sensitivity to chemotherapy drugs [20]. Mardilovich et al. found that the LIMK1 level was elevated in non-metastatic prostate cancer [21]. Overexpression of LIMK1 promotes the migration of multidrug-resistant osteosarcoma cells [22]. Several other independent studies have also reported that LIMK1 expression is closely associated with many kinds of cancers.

However, the effects of LIMK1 on OSCC are still poorly understood. In this study, the expression of LIMK1 was found to be significantly higher in OSCC tissues and cell lines than in the corresponding healthy tissue. Moreover, inhibition of LIMK1 could dramatically suppress the proliferation and EMT of OSCC cells.

LIMK1 has only one known physiological substrate: CFL1, a small protein that is required for tumor cell movement [23]. During mitosis and/or meiosis, the LIMKs-CFL1 pathway may function as an important regulator of actin cytoskeletal rearrangements, chromosome segregation, and cytokinesis (REF). This pathway is also involved in cell cycle regulation [24].

More and more studies have shown that miRNAs can act as tumor regulators, either as cancer suppressors or oncogenes [25, 26]. For example, Pan et al. discovered that miR-106a inhibited the cell migration and invasion of renal cell carcinoma by regulating PAK5 expression [27]. Hao et al. found that miR-106a suppressed tumor cells death in colorectal cancer by directly targeting ATG7 [28]. Decreased serum levels of miR-106a were associated with high grade prostate cancer and high risk of recurrence and progression. [29]. However, it has also been reported miR-106 functions as an oncogene in human gastric cancer, and it contributes to proliferation and metastasis in vitro and in vivo [30]. Xie et al. showed that miR-106a promoted the growth and metastasis of non-small cell lung cancer by targeting PTEN [31]. The levels of miR-106a were also significantly upregulated in high-grade, high-risk and non-muscle invasive bladder cancer [32].

In this study, we found that the level of miR-106a was significantly lower in OSCC tissues than in the adjacent normal tissues. We also demonstrated that miR-106a was downregulated in OSCC cell lines compared to NHOK cells. When we studied the roles of miR-106a in OSCC cells, we confirmed that the level of miR-106a can be regulated in OSCC cells and found that upregulation of miR-106a dramatically inhibited OSCC cell proliferation and EMT compared to the cells transfected with miR-NC.

Multiple studies have confirmed that miRNAs could regulate the expression of LIMK1 and affect its biological function in different cancer types. For example, miR-20a inhibits cutaneous squamous cell carcinoma metastasis and proliferation by directly targeting LIMK1 [33]. MiR-143 inhibits NSCLC cell growth and metastasis through directly regulating LIMK1 [34]. MiR-138 inhibits migration and invasion of NSCLC cells by targeting LIMK1 [35]. However, no previous studies demonstrated a relationship between miR-106a and LIMK1 in OSCC.

We applied the luciferase reporter assay to confirm that LIMK1 might be a target gene of miR-106a in OSCC cells. Both the qRT-PCR and western blot assays showed that the level of LIMK1 can be negatively regulated by miR-106a, which played a role by binding with a site in the LIMK1 3’-UTR. Previous reports have shown that other genes, such as Sox2, SIRT7 and Semaphorin-7A, have also demonstrated the effects on EMT in OSCC [36–38]. In this study, we transfected LIMK1 into miR-106a-overexpressing cells. The results showed that overexpression of LIMK1 led to the recovery of the proliferation and EMT of OSCC cells suppressed by upregulation of miR-106a. These results illustrated that miR-106a might act as a tumor suppressor in OSCC by targeting LIMK1.

## Conclusions

Our results show that the expression of LIMK1 was significantly upregulated and miR-106a level was dramatically downregulated in OSCC tissues. Overexpression of miR-106a inhibited the invasion and EMT of OSCC cells through direct downregulation of LIMK1 expression. Therefore, our study provides functional evidence to support the hypothesis that miR-106a and LIMK1 are prognostic factors for OSCC.

## Abbreviations

EMT: Epithelial–mesenchymal transition
LIMK1: LIM kinase 1
miRNA: microRNA
MMP: Matrix metalloproteinase
OSCC: Oral squamous cell carcinoma

## References

[1] Perez-Sayans M, Somoza-Martin JM, Barros-Angueira F, Reboiras-Lopez MD, Gandara Rey JM, Garcia-Garcia A (2009). Genetic and molecular alterations associated with oral squamous cell cancer (review). Oncol Rep.

[2] Lwin CT, Hanlon R, Lowe D, Brown JS, Woolgar JA, Triantafyllou A, Rogers SN, Bekiroglu F, Lewis-Jones H, Wieshmann H, Shaw RJ (2012). Accuracy of MRI in prediction of tumour thickness and nodal stage in oral squamous cell carcinoma. Oral Oncol.

[3] Jensen DH, Dabelsteen E, Specht L, Fiehn AM, Therkildsen MH, Jonson L, Vikesaa J, Nielsen FC, von Buchwald C (2015). Molecular profiling of tumour budding implicates TGFbeta-mediated epithelial-mesenchymal transition as a therapeutic target in oral squamous cell carcinoma. J Pathol.

[4] Noguti J, De Moura CF, De Jesus GP, Da Silva VH, Hossaka TA, Oshima CT, Ribeiro DA (2012). Metastasis from oral cancer: an overview. Cancer Genomics Proteomics.

[5] Patel SG, Amit M, Yen TC, Liao CT, Chaturvedi P, Agarwal JP, Kowalski LP, Ebrahimi A, Clark JR, Cernea CR, Brandao SJ, Kreppel M, Zoller J, Fliss D, Fridman E, Bachar G, Shpitzer T, Bolzoni VA, Patel PR, Jonnalagadda S, Robbins KT, Shah JP, Gil Z (2013). Lymph node density in oral cavity cancer: results of the international consortium for outcomes research. Br J Cancer.

[6] Mardilovich K, Baugh M, Crighton D, Kowalczyk D, Gabrielsen M, Munro J, Croft DR, Lourenco F, James D, Kalna G, McGarry L, Rath O, Shanks E, Garnett MJ, McDermott U, Brookfield J, Charles M, Hammonds T, Olson MF (2015). LIM kinase inhibitors disrupt mitotic microtubule organization and impair tumor cell proliferation. Oncotarget.

[7] Su J, Zhou Y, Pan Z, Shi L, Yang J, Liao A, Liao Q, Su Q. Downregulation of LIMK1-ADF/cofilin by DADS inhibits the migration and invasion of colon cancer. Sci Rep. 2017;7:45624. 10.1038/srep45624. 10.1038/srep45624 PMC5372356 28358024

[8] Su B, Su J, Zeng Y, Liu F, Xia H, Ma YH, Zhou ZG, Zhang S, Yang BM, Wu YH, Zeng X, Ai XH, Ling H, Jiang H, Su Q (2016). Diallyl disulfide suppresses epithelial-mesenchymal transition, invasion and proliferation by downregulation of LIMK1 in gastric cancer. Oncotarget.

[9] You T, Gao W, Wei J, Jin X, Zhao Z, Wang C, Li Y (2015). Overexpression of LIMK1 promotes tumor growth and metastasis in gastric cancer. Biomed Pharmacother.

[10] Zheng B, Liang L, Wang C, Huang S, Cao X, Zha R, Liu L, Jia D, Tian Q, Wu J, Ye Y, Wang Q, Long Z, Zhou Y, Du C, He X, Shi Y (2011). MicroRNA-148a suppresses tumor cell invasion and me tastasis by downregulating ROCK1 in gastric cancer. Clin Cancer Res.

[11] Yu X, Li Z, Shen J, Wu WK, Liang J, Weng X, Qiu G (2013). MicroRNA-10b promotes nucleus pulposus cell proliferation through RhoC-Akt pathway by targeting HOXD10 in intervetebral disc degeneration. PLoS One.

[12] Yu X, Li Z, Chen G, Wu WK (2015). MicroRNA-10b induces vascular muscle cell proliferation through Akt pathway by targeting TIP30. Curr Vasc Pharmacol.

[13] Liu G, Cao P, Chen H, Yuan W, Wang J, Tang X (2013). MiR-27a regulates apoptosis in nucleus pulposus cells by targeting PI3K. PLoS One.

[14] Song Q, Xu Y, Yang C, Chen Z, Jia C, Chen J, Zhang Y, Lai P, Fan X, Zhou X, Lin J, Li M, Ma W, Luo S, Bai X (2014). miR-483-5p promotes invasion and metastasis of lung adenocarcinoma by targeting RhoGDI1 and ALCAM. Cancer Res.

[15] Wan L, Zhang L, Fan K, Wang J (2014). MiR-27b targets LIMK1 to inhibit growth and invasion of NSCLC cells. Mol Cell Biochem.

[16] Li Z, Yu X, Wang Y, Shen J, Wu WK, Liang J, Feng F (2015). By downregulating TIAM1 expression, microRNA-329 suppresses gastric cancer invasion and growth. Oncotarget.

[17] Yu X, Li Z (2016). The role of miRNAs in cutaneous squamous cell carcinoma. J Cell Mol Med.

[18] Li Z, Lei H, Luo M, Wang Y, Dong L, Ma Y, Liu C, Song W, Wang F, Zhang J, Shen J, Yu J (2015). DNA methylation downregulated mir-10b acts as a tumor suppressor in gastric cancer. Gastric Cancer.

[19] Liao Q, Li R, Zhou R, Pan Z, Xu L, Ding Y, Zhao L (2017). LIM kinase 1 interacts with myosin-9 and alpha-actinin-4 and promotes colorectal cancer progression. Br J Cancer.

[20] Chen Q, Jiao D, Hu H, Song J, Yan J, Wu L (2013). Xu LQ. Downregulation of LIMK1 level inhibits migration of lung cancer cells and enhances sensitivity to chemotherapy drugs. Oncol Res.

[21] Mardilovich K, Gabrielsen M, McGarry L, Orange C, Patel R, Shanks E, Edwards J, Olson MF (2015). Elevated LIM kinase 1 in nonmetastatic prostate cancer reflects its role in facilitating androgen receptor nuclear translocation. Mol Cancer Ther.

[22] Zhang H, Wang Y, Xing F, Wang J, Wang Y, Wang H, Yang Y, Gao Z (2011). Overexpression of LIMK1 promotes migration ability of multidrug-resistant osteosarcoma cells. Oncol Res.

[23] Cai S, Chen R, Li X, Cai Y, Ye Z, Li S, Li J, Huang H, Peng S, Wang J, Tao Y, Huang H, Wen X, Mo J, Deng Z, Wang J, Zhang Y, Gao X, Wen X (2015). Downregulation of microRNA-23a suppresses prostate cancer metastasis by targeting the PAK6-LIMK1 signaling pathway. Oncotarget.

[24] Meng Y, Takahashi H, Meng J, Zhang Y, Lu G, Asrar S, Nakamura T, Jia Z (2004). Regulation of ADF/cofilin phosphorylation and synaptic function by LIM-kinase. Neuropharmacology.

[25] Barger JF, Nana-Sinkam SP (2015). MicroRNA as tools and therapeutics in lung cancer. Respir Med.

[26] Kang SM, Lee HJ (2014). MicroRNAs in human lung cancer. Exp Biol Med (Maywood).

[27] Pan YJ, Wei LL, Wu XJ, Huo FC, Mou J, Pei DS (2017). MiR-106a-5p inhibits the cell migration and invasion of renal cell carcinoma through targeting PAK5. Cell Death Dis.

[28] Hao H, Xia G, Wang C, Zhong F, Liu L, Zhang D (2017). miR-106a suppresses tumor cells death in colorectal cancer through targeting ATG7. Med Mol Morphol.

[29] Cochetti G, Poli G, Guelfi G, Boni A, Egidi MG, Mearini E (2016). Different levels of serum microRNAs in prostate cancer and benign prostatic hyperplasia: evaluation of potential diagnostic and prognostic role. Onco Targets Ther..

[30] Zhu M, Zhang N, He S, Yan R, Zhang J (2016). MicroRNA-106a functions as an oncogene in human gastric cancer and contributes to proliferation and metastasis in vitro and in vivo. Clin Exp Metastasis.

[31] Xie X, Liu HT, Mei J, Ding FB, Xiao HB, Hu FQ, Hu R, Wang MS (2015). miR-106a promotes growth and metastasis of non-small cell lung cancer by targeting PTEN. Int J Clin Exp Pathol.

[32] Mearini E, Poli G, Cochetti G, Boni A, Egidi MG, Brancorsini S (2017). Expression of urinary miRNAs targeting NLRs inflammasomes in bladder cancer. Onco Targets Ther.

[33] Zhou J, Liu R, Luo C, Zhou X, Xia K, Chen X, Zhou M, Zou Q, Cao P, Cao K (2014). MiR-20a inhibits cutaneous squamous cell carcinoma metastasis and proliferation by directly targeting LIMK1. Cancer Biol Ther.

[34] Xia H, Sun S, Wang B, Wang T, Liang C, Li G, Huang C, Qi D, Chu X (2014). miR-143 inhibits NSCLC cell growth and metastasis by targeting Limk1. Int J Mol Sci.

[35] Tan Y, Hu H, Tan W, Jin L, Liu J, Zhou H (2016). MicroRNA-138 inhibits migration and invasion of non-small cell lung cancer cells by targeting LIMK1. Mol Med Rep.

[36] Liu X, Qiao B, Zhao T, Hu F, Lam AK, Tao Q (2018). Sox2 promotes tumor aggressiveness and epithelial-mesenchymal transition in tongue squamous cell carcinoma. Int J Mol Med.

[37] Li W, Zhu D (2018). SIRT7 suppresses the epithelial-to-mesenchymal transition in oral squamous cell carcinoma metastasis by promoting SMAD4 deacetylation. J Exp Clin Cancer Res.

[38] Liu TJ, Guo JL, Wang HK, Xu X (2018). Semaphorin-7A contributes to growth, migration and invasion of oral tongue squamous cell carcinoma through TGF-β-mediated EMT signaling pathway. Eur Rev Med Pharmacol Sci.

